# The effect of increased positive end expiratory pressure on brain tissue oxygenation and intracranial pressure in acute brain injury patients

**DOI:** 10.1038/s41598-023-43703-9

**Published:** 2023-10-03

**Authors:** Elisa Gouvea Bogossian, Joaquin Cantos, Anita Farinella, Leda Nobile, Hassane Njimi, Giacomo Coppalini, Alberto Diosdado, Michele Salvagno, Fernando Oliveira Gomes, Sophie Schuind, Marco Anderloni, Chiara Robba, Fabio Silvio Taccone

**Affiliations:** 1 Department of Intensive Care, Hôpital Universitaire de Bruxelles (HUB), Université Libre de Bruxelles (ULB), Erasme Hospital, Université Libre de Bruxelles, Route de Lennik, 808, 1070 Brussels, Belgium; 2 Critical Care Department, Hospital Italiano de Buenos Aires, Buenos Aires, Argentina; 3 Department of Neurosurgery, Hôpital Universitaire de Bruxelles (HUB), Université Libre de Bruxelles (ULB), Université Libre de Bruxelles, Brussels, Belgium; 4 Dipartimento di Scienze Chirurgiche e Diagnostiche, IRCCS Policlinico San Martino, Università di Genova, Genova, Italy

**Keywords:** Neurological disorders, Brain injuries, Cerebrovascular disorders, Stroke

## Abstract

Cerebral hypoxia is an important cause of secondary brain injury. Improving systemic oxygenation may increase brain tissue oxygenation (PbtO_2_). The effects of increased positive end-expiratory pressure (PEEP) on PbtO_2_ and intracranial pressure (ICP) needs to be further elucidated. This is a single center retrospective cohort study (2016–2021) conducted in a 34-bed Department of Intensive Care unit. All patients with acute brain injury under mechanical ventilation who were monitored with intracranial pressure and brain tissue oxygenation (PbtO_2_) catheters and underwent at least one PEEP increment were included in the study. Primary outcome was the rate of PbtO_2_ responders (increase in PbtO_2_ > 20% of baseline) after PEEP increase. ΔPEEP was defined as the difference between PEEP at 1 h and PEEP at baseline; similarly ΔPbtO_2_ was defined as the difference between PbtO_2_ at 1 h after PEEP incrementation and PbtO_2_ at baseline. We included 112 patients who underwent 295 episodes of PEEP increase. Overall, the median PEEP increased form 6 (IQR 5–8) to 10 (IQR 8–12) cmH_2_O (p = 0.001), the median PbtO_2_ increased from 21 (IQR 16–29) mmHg to 23 (IQR 18–30) mmHg (p = 0.001), while ICP remained unchanged [from 12 (7–18) mmHg to 12 (7–17) mmHg; p = 0.42]. Of 163 episode of PEEP increments with concomitant PbtO_2_ monitoring, 34 (21%) were PbtO_2_ responders. A lower baseline PbtO_2_ (OR 0.83 [0.73–0.96)]) was associated with the probability of being responder. ICP increased in 142/295 episodes of PEEP increments (58%); no baseline variable was able to identify this response. In PbtO_2_ responders there was a moderate positive correlation between ΔPbtO_2_ and ΔPEEP (r = 0.459 [95% CI 0.133–0.696]. The response in PbtO_2_ and ICP to PEEP elevations in brain injury patients is highly variable. Lower PbtO_2_ values at baseline could predict a significant increase in brain oxygenation after PEEP increase.

## Introduction

Prevention, identification and early treatment of secondary brain injury is an important aspect of the management of acute brain injury (ABI) in order to improve outcome^1^. Cerebral hypoxia is an important cause of secondary brain injury and can frequently affect ABI patients with both traumatic and non-traumatic etiology^2–6^. Indeed, low brain tissue partial pressure of oxygen (PbtO_2_) has been associated with cerebral anaerobic metabolism, increased risk of mortality and poor functional outcome^7–13^.

PbtO_2_ is a focal measurement of brain oxygenation reflecting an equilibrium between oxygen delivery (i.e. cerebral blood flow, CBF, hemoglobin and arterial oxygenation), consumption (i.e. brain metabolism, mitochondrial function and body temperature) and extraction (i.e. microcirculation and mitochondrial function)^14,15^. Therefore, different pathophysiological mechanisms are associated with low PbtO_2_ after acute brain injury, such as reduced CBF and/or cerebral perfusion pressure (CPP), intracranial hypertension (IH), hypoxemia, anemia, altered microcirculation or excessive cellular metabolism^16^. Hypoxemia itself has also been associated with poor outcomes after traumatic brain injury^17,18^. Therefore, strategies aiming at improving hypoxemia may be useful in this setting_._

In patient undergoing mechanical ventilation, applying a positive end-expiratory pressure (PEEP) has been shown to reduce end-expiratory alveolar collapse, maintaining alveolar recruitment and possibly reduce the incidence of ventilator-associated pneumonia and lung injury^19–22^. These effects are more pronounced in patients with acute respiratory distress syndrome (ARDS)^23,24^. By improving systemic oxygenation, increased PEEP could improve brain tissue oxygenation. However, the existing literature is scarce. Small studies have shown an increase in PbtO_2_ following increases in PEEP and recruitment manouvers^25,26^, while others have not^27^. Moreover, while PEEP may increase PbtO_2_ by increasing systemic oxygenation, it can potentially also increase ICP, by reducing cerebral venous outflow due to increased intrathoracic pressure^28^, or decrease mean arterial pressure (MAP), by impairing venous return to the heart^27,29^.

Therefore, the aim of this study was to assess the impact of increase in PEEP levels on PbtO_2_ and ICP in ABI patients. The secondary aim was to identify baseline factors associated with PbtO_2_ increase and ICP increase.

## Methods

### Study design

This was a single center retrospective cohort study conducted at the Intensive Care Unit (ICU) of the Hôpital Universitaire de Bruxelles (HUB), in Brussels, Belgium, from January 2016 to December 2021. The study protocol was approved by Erasme Hospital Ethics Committee (P2022/449) and the need for informed written consent was waived, because of the anonymized data collection of retrospective data. This study was carried out in accordance with the Strengthening The Reporting of Observational studies in Epidemiology (STROBE) statement^30^.

### Study population

We screened adult (> 18 years) patients admitted to the ICU due to an acute brain injury. Inclusion criteria were: (a) traumatic brain injury (TBI) or aneurysmal subarachnoid hemorrhage (aSAH) patients under controlled mechanical ventilation; (b) the presence of ICP and PbtO_2_ monitoring within the first 48 h of admission and (c) patients underwent at least one increase in PEEP levels concomitantly with the use of neuromonitoring. Patients with imminent death were excluded.

### Patients’ management

We followed the current guidelines for the management of TBI^31^ and SAH^32^; invasive multimodal monitoring, including PbtO_2_, was implemented according to recent consensus^1^. PbtO_2_ monitoring is considered as “standard of care” for ABI patients with a Glasgow Coma Score (GCS) < 9 and requiring intracranial pressure (ICP) monitoring; PbtO_2_ probes were placed in the frontal region of the hemisphere at greatest risk for secondary brain injury. In TBI patients, probe was placed close < 5 cm to the most injured/contused area in TBI; in SAH patients probes were placed in the region at risk or with demonstrated delayed cerebral ischemia for aSAH). Probes were inserted through a frontal burr hole using a triple-lumen bolt. PbtO_2_ was monitored continuously using a specific probe and the Integra Licox® Brain Tissue Oxygen Monitoring System (IM3.ST_EU, Integra LifeSciences Corporation, Plainsboro, NJ, USA). Probe location was confirmed by a cerebral CT-scan performed within 24 h from neuromonitoring placement. The adequate functioning of the probe was tested with a 100% oxygen fraction (FiO_2_) test for 15 min (i.e. an increase of at least 5 mmHg of PbtO_2_ indicated an adequate catheter function)_._ Intracranial pressure probes were placed either intraventricular or intraparenchymal.

#### PEEP trials

PEEP was increased according to the decision of the critical care team responsible for the treatment of the patient. There was no standardized protocol. However, PEEP was increased in increments of 2 mmHg for at least 1 h-aiming at improving systemic oxygenation (targeting an improvement in arterial partial pressure of oxygen and inspired fraction of oxygen ration) and respiratory mechanics while minimizing ventilator induced lung injury^33^ by implementing a lung protective ventilation strategy and targeting Plateau pressure < 30 mmHg and driving pressure < 15 mmHg^34^. If after one 1 h of PEEP incrementation there was no benefit in oxygenation or if there were deleterious effects on respiratory mechanics or persistent hemodynamic instability, PEEP was reversed to baseline setting. The decision on the amount of PEEP to incrementation was done by the respiratory therapist in agreement with the treatment treating physician. No FiO_2_ changes were implemented during the PEEP trial.

### Data collection

Physiological variables, ICP and PbtO_2_ were measured in real-time and collected prospectively on a patient data monitoring system. Cerebral perfusion pressure (CPP) was calculated as the difference between MAP and ICP; MAP was zeroed at the level of the left atrium. Intracranial hypertension was defined as ICP value above 20 mmHg for at least 5 min at any time. Brain tissue hypoxia was defined as a PbtO_2_ < 20 mmHg for at least 5 min.

Baseline (T0) was defined as the hour immediately preceding a PEEP change and T1 was defined as the first 1 h with the new stable PEEP value. The 60-min mean of value of the following continuous variables was collected at T0 and T1: mean arterial pressure (MAP), heart rate (HR), ICP, CPP and PbtO_2_. ICP and PbtO_2_ were recorded prospectively every minute. Data was extracted from the monitoring system and the mean value of ICP and PbtO_2_ was calculated for every hour of monitoring.

ICP and PbtO_2_ changes (ΔICP and ΔPbtO_2_) were calculated as the difference between ICP or PbtO_2_ at T1 and T0. A “decrease” in ICP or PbtO_2_ was identified as a ΔICP/ΔPbtO_2_ < 0; a “stable” value as a ΔICP/ΔPbtO_2_ = 0 and an “increase” as a ΔICP/ΔPbtO_2_ > 0. Patients with a PbtO_2_ increase of more than 20% from baseline were considered as “responders”; a significant increase in ICP was defined as an increase of more than 20% from baseline or an increase that led to intracranial hypertension. The following ventilator parameters were recorded at T0 and T1: inspiratory pressure (Pins); tidal volume (V_T_), PEEP; respiratory rate (RR) and inspired fraction of oxygen (FiO_2_). Arterial oxygen saturation (SaO_2_), lactate, pH, PaCO_2_, PaO_2_ were also recorded.

We collected demographics, the presence of comorbidities, sequential organ failure assessment (SOFA)^35^ and the Glasgow coma scale (GCS)^36^ on admission. Hospital mortality and the Glasgow Outcome Scale (GOS)^37^ at 3 months were collected, as previously reported^38^. Unfavorable neurological outcome (UO) was defined as GOS of 1–3.

### Study outcomes

The primary outcome was the proportion of PbtO_2_ responders. Secondary outcomes included: (a) the proportion of patients with an increase of ICP > 20% or an increase in ICP that resulted in intracranial hypertension after PEEP augmentation; (b) the correlation between the difference of PEEP at T1 and T0 (ΔPEEP) and ΔICP/ΔPbtO_2_; (c) baseline factors associated with significant increases in PbtO_2_ and ICP after PEEP augmentation; (d) the proportion of patients with an decrease in CPP resulting on a CPP < 60 mmHg; (e) differences between the trends in PbtO_2_ and ICP during PEP incrementation challenge in SAH and TBI patients.

### Statistical analysis

Descriptive statistics were computed for all variables. Categorical variables were described as proportions (%) and compared using Chi square or Fisher’s exact test. Normality was assessed using the Kolmogorov–Smirnov test. Normally distributed variables were expressed as mean (± SD) and compared using Student t test while non-gaussian continuous variables were described median [IQRs] and compared using Mann–Whitney test (independent variables) or Wilcoxon test (repeated measures of related variables). A Spearman correlation was computed between ΔPEEP_,_ ΔPbtO2, ΔICP, ΔCPP and ΔPaO_2_. As a sensitivity analysis we also considered just the first PEEP increment of each patient to calculate the correlation between ΔPEEP_,_ ΔPbtO_2_, ΔICP, ΔCPP and ΔPaO_2_. To account for multiple measures per patient a generalized mixed model with logit link was used to identify baseline variables which were independently associated with a PbtO_2_ responder and a significant increase in ICP after PEEP increment; baseline variables with a p value < 0.1 in the univariate analysis were included in the multivariable analysis. Odds ratios (ORs) with 95% confidence intervals (CIs) were computed for all variables. The independence of errors, presence of multicollinearity and of influential outlier assumptions were checked; none were violated. A receiver operator curve was designed to assess the sensitivity and specificity of baseline PbtO_2_ to identify responders. The area under the curve (AUROC) and CI 95% were computed. Youden’s test was used to identify the cut-off with the best sensitivity and specificity. We used a similar model to identify variables associated with an absolute increase in PbtO_2_ and ICP after PEEP increments. All statistical analyses were performed using SPSS 27.0 for MacIntosh. A p value < 0.05 was considered significant.

### Ethics approval and consent to participate

The study protocol was approved by local ethics Committees (Erasme Hospital: P2022/449) and informed written consent was waived. All methods were carried out in accordance with relevant scientific and ethical guidelines and regulations.

## Results

### Study population

**Figure 1:**
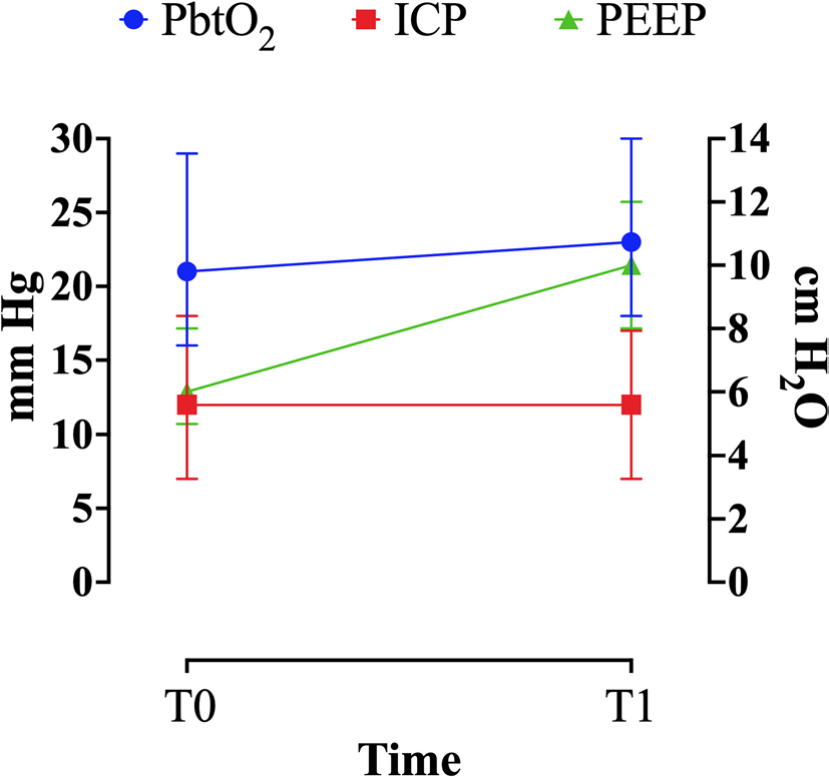
Variations of intracranial hypertension and of brain tissue oxygenation (PbtO2) before (T0) and after (T1) increments in positive end expiratory pressure (PEEP). PEEP changed from 6 cmH_2_O (5–8) to 10 cmH_2_O (8–12), p = 0.001. ICP varied from 12 mmHg (7–18) to 12 mmHg (7–17), p = 0.42. PbtO_2_ changed from 21 mmHg (16–29) to 23 mmHg (18–30), p = 0.001.

Over a total of 237 patients monitored with ICP and PbtO_2_ during the study period, 112 patients (TBI, n = 47 [42%]; SAH, n = 65 [58%]) had an increase in PEEP values under controlled mechanical ventilation (a total of 295 PEEP increase for ICP and 163 for PbtO_2_, as in some patients PbtO_2_ catheter was removed). The characteristics of the population are shown in Table 1. Mean age and median GCS on admission were 51(± 15) years and 5 (3–9), respectively. During the ICU stay, intracranial hypertension occurred in 89/112 patients (79%) and brain tissue hypoxia occurred in 94/112 (84%). A total of 59 (53%) of patients died in the hospital and 97 (87%) had unfavorable outcome. Table 2 shows ventilatory parameters and physiological variables at T0 and T1; PEEP was increased from 6 (5–8) to 10 (8–12) cmH_2_O (p = 0.001); PbtO_2_ significantly increased from 21 (16–29) to 23 (18–30) mmHg (p = 0.001), while ICP remained stable over time, varying from 12 (7–18) to 12 (7–17) mmHg (p = 0.42), as shown in Fig. 1. During the same time-period, MAP and CPP remained unchanged.

**Table 1:** Characteristics of the study population.

	All patients (N = 112)
Age, years—median (IQR)	51 (± 15)
Male gender, n (%)	59 (53)
Glasgow coma scale on admission—median (IQR)	5 (3–9)
SOFA score at ICU admission—median (IQR)	9 (6–10)
Underlying disease, n (%)	
Non-traumatic SAH	65 (58)
WFNS 4–5	48 (74%)
mFisher	4 (4–4)
Traumatic brain injury	47 (42)
Marshall score	
0	0
1	0
2	1 (2)
3	0
4	2 (4)
5	44 (94)
Comorbidities	
Arterial hypertension, n (%)	39 (35)
Chronic heart disease (NYHA 3 or 4), n (%)	17 (15)
Diabetes mellitus, n (%)	12 (11)
COPD, n (%)	11 (10)
Chronic kidney disease, n (%)	2 (2)
Liver cirrhosis, n (%)	4 (4)
Cancer, n (%)	7 (6)
Previous neurological disease, n (%)	10 (9)
During ICU stay	
Mechanical ventilation, n (%)	112 (100)
Vasopressors, n (%)	107 (96)
Inotropes, n (%)	40 (36)
RRT, n (%)	1 (1)
ECMO, n (%)	4 (4)
Neurological complications	
Intracranial hypertension, n (%)	89 (80)
Brain tissue hypoxia, n (%)	94 (82)
Seizures, n (%)	32 (29)
Outcomes	
ICU LOS, days—median (IQR)	18 (9–26)
Hospital LOS, days—median (IQR)	26 (10–63)
ICU death, (%)	57 (51)
Hospital death, n (%)	59 (53)
GOS at 3 months—median (IQR)	1 (1–3)
Unfavorable neurological outcome at 3 months, n (%)	97 (87)

*SOFA* sequential organ failure assessment, *WFNS* world federation of neurological surgeons, *NYHA* new york heart association functional classification, *COPD* chronic obstructive pulmonary disease, *RRT* renal replacement therapy, *ECMO* extra corporeal membrane oxygenation, *ICU* intensive care unit, *GOS* Glasgow outcome scale, *LOS* length of stay.

**Table 2:** Ventilatory and physiological variables at T0 and T1.

	T0	T1	P value
Vt (mL)	450 (400–478)	450 (390–470)	0.12
Pinsp (cmH_2_O)	8 (6–10)	8 (6–10)	0.86
PEEP (cmH_2_O)	6 (5–8)	10 (8–12)	0.001
RR (breaths per minute)	24 (19–28)	24 (18–28)	0.24
FiO_2_	0.50 (0.35–0.60)	0.50 (0.40–0.60)	0.78
MAP (mmHg)	104 (93–117)	104 (91–119)	0.64
ICP (mmHg)	12 (7–18)	12 (7–17)	0.42
CPP (mmHg)	91 (79–105)	91 (76–105)	0.47
PbtO_2_ (mmHg)	21 (16–29)	23 (18–30)	0.001
P/F	192 (141–273)	206 (143–284)	0.76
PaCO_2_ (mmHg)	40 (36–45)	41 (37–47)	0.52

Data are expressed as median and interquartile range.

*Vt* tidal volume, *Pinsp* inspiratory pressure, *PEEP* positive end expiratory pressure, *RR* respiratory rate, *FiO2* inspired fraction of oxygen, *MAP* mean arterial pressure, *ICP* intracranial pressure, *CPP* cerebral perfusion pressure, *PbtO*_*2*_ brain tissue partial pressure of oxygen, *P/F* arterial partial pressure of oxygen/ inspired fraction of oxygen, *PaCO*_*2*_ arterial partial pressure of carbon dioxide.

### PEEP augmentation and PbtO_2_ values

There was an absolute increase in PbtO_2_ in 102/163 (63%) episodes of PEEP augmentation; in 30 (18%) episodes, there were no changes in PbtO_2_, while in 31 (19%) episodes there was a decrease in PbtO_2_. Baseline PbtO_2_ was below 20 mmHg in 63/163 (39%) episodes. Higher baseline PaO_2_ (OR 1.02 [95% CI 1.01–1.03]) and lower baseline PbtO_2_ (OR 0.92 [95% CI 0.91–0.94]) were independently associated with any increase in PbtO_2_ after PEEP augmentation (Supplemental Tables S1 and S2).

There was no correlation between ΔPEEP and ΔPbtO_2_ (r = 0.084 [95% CI − 0.075 to 0.24], Supplemental Fig. S1A), even when we considered only the first measure per patient (r = 0.197 [95% CI − 0.100 to 0.425], Supplemental Fig. S1B). Considering all measurements, there was a weak correlation between ΔPbtO_2_ and ΔPaO_2_ (r = 0.223 [95% CI 0.030 to 0.400]); considering only the first measure per patient, a weak correlation between ΔPbtO_2_ and ΔPaO_2_ was observed (r = 0.275 [95% CI 0.0254 to 0.530]).

### PbtO_2_ responders

**Figure 2:**
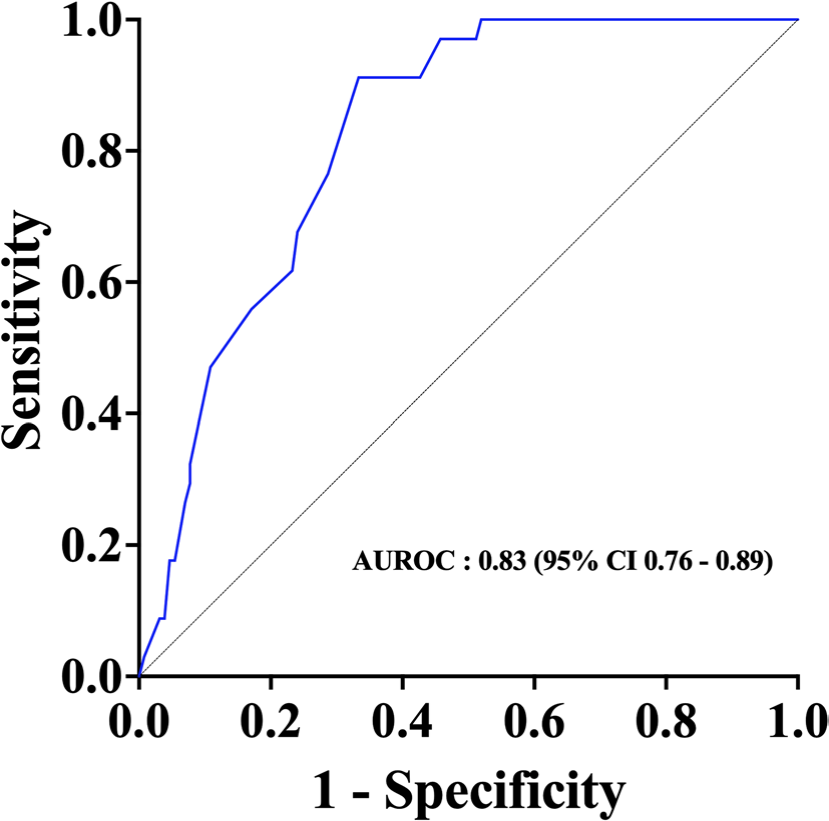
Receiver operator curve of baseline PbtO_2_ and responders.

We identified 34/163 (21%) PbtO_2_ responders after PEEP incrementation; lower levels of baseline PbtO_2_ were associated with being a responder (Table 3: OR 0.83 95% CI 0.73–0.95, p = 0.009). Baseline PbtO2 had an AUROC of 0.83 (0.76–0.89) to predict PbtO2 responder after PEEP augmentation (Fig. 2); the baseline PbtO_2_ cut-off with the best sensitivity (91.2%) and specificity (66.7%) was 21 mmHg.

**Table 3:** Univariable generalized mixed model for fixed effects logit link function to assess the impact of baseline variables on the significant increase (responders) in brain tissue oxygenation (PbtO_2_) after positive end expiratory pressure (PEEP) increments.

	Univariable analysis OR (95% CI)	P value
Age, year	1.004 (0.977–1.031)	0.79
Male sex	0.832 (0.341–2.032)	0.69
Glasgow Coma Scale on admission	1.001 (0.892–1.144)	0.88
SOFA score at ICU admission	1.087 (0.955–1.237)	0.21
TBI compared to SAH	1.010 (0.350–2.915)	0.99
Baseline PEEP	1.024 (0.915–1.145)	0.69
Baseline ICP	0.957 (0.907–1.011)	0.11
Baseline CPP	1.014 (0.986–1.043)	0.33
Baseline PbtO_2_	0.834 (0.728–0.955)	0.009
Baseline PaO_2_	1.008 (0.993–1.024)	0.29
Baseline PaCO_2_	0.986 (0.930–1.046)	0.65

Data are expressed as odds ratio and 95% confidence intervals. Data from 112 patients with 163 episodes of PEEP incrementation were included in this analysis.

*SOFA* sequential organ failure assessment, *ICU* intensive care unit, *TBI* traumatic brain injury, *SAH* subarachnoid hemorrhage, *PEEP* positive end expiratory pressure, *ICP* intracranial pressure, *CPP* cerebral perfusion pressure, *PbtO*_*2*_ brain tissue partial pressure of oxygen, *PaO*_*2*_ arterial partial pressure of oxygen, *PaCO*_*2*_ arterial partial pressure of carbon dioxide.

**Figure 3:**
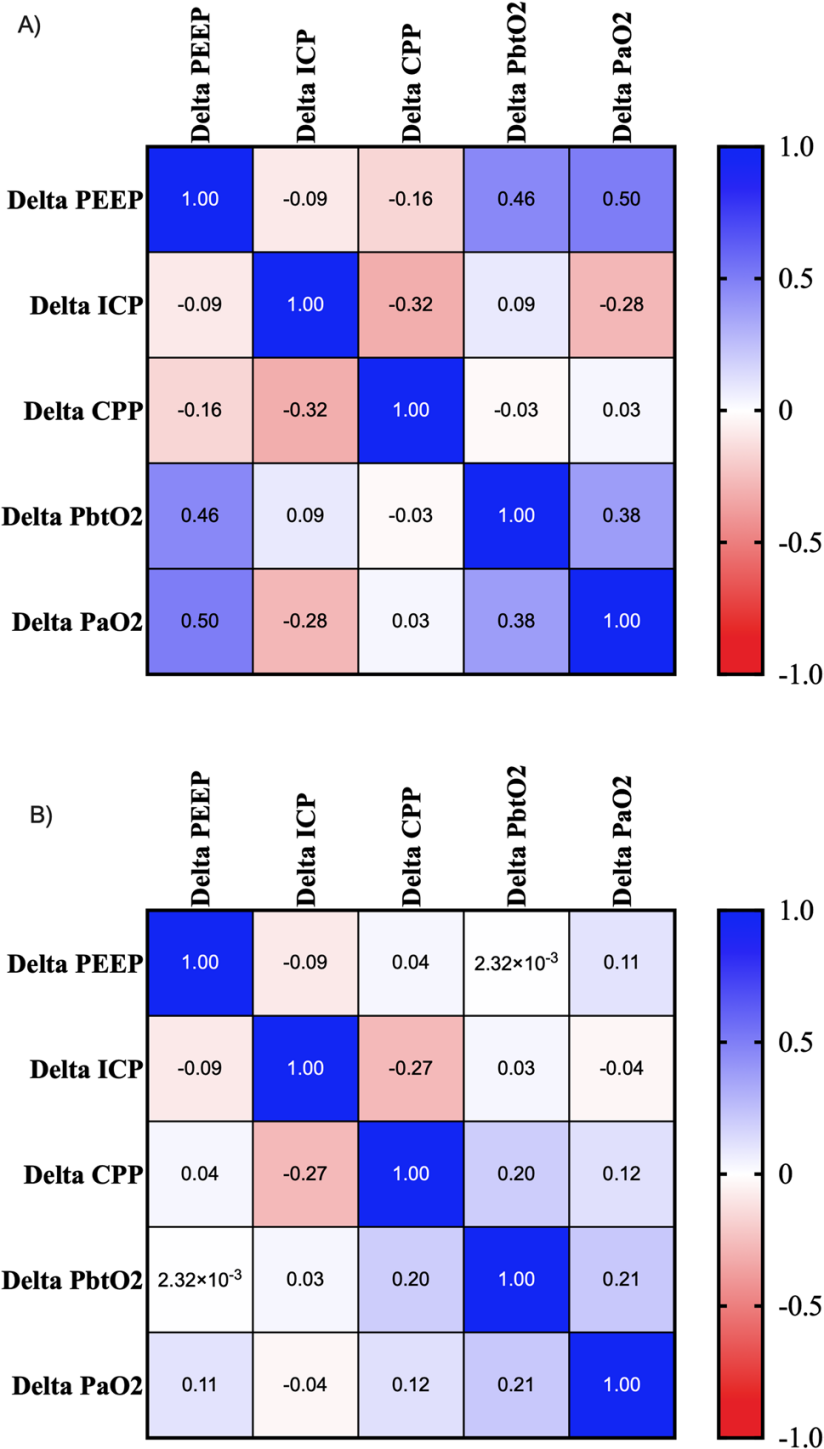
Correlation matrix analyzed by the Spearman method between changes in PEEP, ICP, PbtO_2_, CPP and PaO_2_. Panel (**A**) PbtO_2_ responders. Panel (**B**) PbtO_2_ non responders.

In PbtO_2_ responders, there was a moderate positive correlation between ΔPbtO_2_ and ΔPEEP (r = 0.459 [95% CI 0.133 to 0.696]), ΔPbtO_2_ and ΔPaO_2_ (r = 0.382 [95% CI 0.001 to 0.672]), while no correlation between ΔPbtO_2_ and ΔCPP was found (r = − 0.027 [95% CI − 0.371 to 0.324]), as shown in Fig. 3A. In non-responders, there was a weak correlation between ΔPbtO_2_ and ΔCPP (r = 0.196 [95% CI 0.019 to 0.361]), ΔPbtO_2_ and ΔPaO_2_ (r = 0.213 [95% CI 0.012 to 0.418]), while no correlation between ΔPbtO_2_ and ΔPEEP was observed (r = 0.002 [95% CI − 0.176 to 0.180]—Fig. 3B).

### PEEP augmentation and ICP values

In 142/295 (48%) episodes of PEEP augmentation, there was an absolute increase in ICP. Baseline ICP was above 20 mmHg in 49/295 episodes (17%). No baseline factors were associated with an absolute ICP increase after PEEP augmentation (Supplemental Tables S3). There was no correlation between ΔPEEP and ΔICP (r = − 0.07 [95% CI − 0.18 to 0.05], Supplemental Fig. S1A), while a moderate inverse correlation between ΔICP and ΔCPP (r = − 0.310 [95% CI − 0.413 to − 0.200]) was observed. When only the first measure per patient was considered, no correlation between ΔPEEP and ΔICP was found (r = − 0.112 [95% − 0.297 to 0.080), while there was a moderate inverse correlation between ΔICP and ΔCPP (r = − 0.403[95% − 0.552 to − 0.230], Supplemental Fig. S1B).

### Patients with significant ICP increase

A significant increase in ICP was observed in 109/295 (37%) episodes of PEEP incrementation. In 23/109 (21%) episodes of significant ICP increase there was an ICP increase > 20% resulting in an ICP > 20 mmHg at T1. No baseline factors were associated with a significant ICP increase (Table 4). In patients with a significant increase in ICP (Supplemental Fig. S2A), no correlation between ΔPEEP and ΔICP (r = − 0.063 [95% CI − 0.53 to 0.132) was observed, but there was a weak inverse correlation between ΔICP and ΔCPP (r = − 0.239 [95% CI − 0.413 to − 0.047). In the other patients (Supplemental Fig. S2B), no correlation between ΔPEEP and ΔICP (r = − 0.039 [95% CI − 0.186 to 0.109) was observed, but there was a weak inverse correlation between ΔICP and ΔCPP (r = − 0.195[95% CI − 0.334 to − 0.049).

**Table 4:** Univariable generalized mixed model for fixed effects logit link function to assess the impact of baseline variables on the a significant increase in ICP (increase of more than 20% of baseline or increased that resulted in ICP > 20 mmHg) after positive end expiratory pressure (PEEP) increments.

	Univariable analysis OR (95% CI)	P value
Age, year	0.998 (0.979–1.018)	0.83
Male sex	1.136 (0.634–2.036)	0.67
Glasgow Coma Scale on admission	1.003 (0.928–1.085)	0.93
SOFA score at ICU admission	1.087 (0.955–1.237)	0.99
TBI compared to SAH	0.614 (0.330–1.144)	0.13
Baseline PEEP	1.039 (0.937–1.152)	0.46
Baseline ICP	0.963 (0.917–1.011)	0.13
Baseline CPP	1.006 (0.990–1.021)	0.47
Baseline PbtO_2_	0.988 (0.960–1.017)	0.40
Baseline PaO_2_	0.998 (0.992–1.004)	0.49
Baseline PaCO_2_	1.023 (0.993–1.054)	0.14

Data are expressed as odds ratio and 95% confidence intervals. Data from 112 patients with 295 episodes of PEEP incrementation were included in this analysis.

*SOFA* sequential organ failure assessment, *ICU* intensive care unit, *TBI* traumatic brain injury, *SAH* subarachnoid hemorrhage, *PEEP* positive end expiratory pressure, *ICP* intracranial pressure, *CPP* cerebral perfusion pressure, *PbtO*_*2*_ brain tissue partial pressure of oxygen, *PaO*_*2*_ arterial partial pressure of oxygen, *PaCO*_*2*_ arterial partial pressure of carbon dioxide.

Of note, in only 4/295 (1.4%) episodes of PEEP incrementation resulted in a CPP of < 60 mmHg.

A significant CPP reduction (T1 CPP < 60 mmhg) was observed in /295.

### Subarachnoid hemorrhage and TBI

In TBI patients (n = 48) PEEP was increased from 5 (5–8) to 10 (8–12) cmH_2_O (p = 0.001); PbtO_2_ significantly increased from 20 (15–25) to 22 (18–28) mmHg (p = 0.001), while ICP did not vary significantly over [from 13 (7–18) to 12 (7–16) mmHg (p = 0.56)]. CPP also remained stable over time [ from 86 (78–95) to 85 (74–95) mmHg (p = 0.50]. There was an absolute increase in PbtO_2_ in 42/59 (71%) episodes of PEEP augmentation; however, only in 13 (22%) episodes there was a significant increase in PbtO_2_ (PbtO_2_ responders). In 11 (17%) episodes, there were no changes in PbtO_2_ while in 6 (10%) episodes there was a decrease in PbtO_2._ In 52/122 (43%) episodes of PEEP augmentation in TBI patients, there was an absolute increase in ICP, while a significant increase in ICP was observed in 38/122 (31%) episodes.

In SAH patients (n = 65) PEEP was increased from 6 (5–8) to 10 (8–12) cmH_2_O (p = 0.001); PbtO_2_ significantly increased from 23 (16–32) to 24 (18–32) mmHg (p = 0.001), while ICP did not vary significantly over [from 12 (7–18) to 12 (7–18) mmHg (p = 0.24)]. CPP also remained stable over time [from 96 (82–110) to 96 (79–110) mmHg (p = 0.73]. There was an absolute increase in PbtO_2_ in 60/104 (58%) episodes of PEEP augmentation; however, we identified only in 21/104 (20%) PbtO_2_ responders. In 19/104 (18%) episodes, there were no changes in PbtO_2_ while in 25/104 (24%) episodes there was a decrease in PbtO_2_. In 52/173 (31%) episodes of PEEP augmentation in SAH patients, there was an absolute increase in ICP, while a significant increase in ICP was observed in 54/122 (26%) episodes.

Patient with TBI and SAH had similar trends regarding PbtO_2_ and ICP variation of time after PEEP challenges as shown in Supplemental Fig. S3*.*

## Discussion

In this study, we observed a significant elevation in PbtO_2_ in 35% of episodes following an increase in PEEP; a lower baseline PbtO_2_ was found to be associated with a significant increase in brain oxygenation. We determined that a baseline PbtO_2_ cut-off of 21 mmHg was optimal for identifying PbtO_2_ responders to PEEP. Additionally, we observed a moderate correlation between changes in PEEP and changes in PbtO_2_, specifically within the group of PbtO_2_ responders. Interestingly, we did not identify any baseline factors associated with a significant increase in ICP, which was observed in 37% cases following PEEP augmentation.

Mechanical ventilation is often required for patients with severe TBI and poor-grade SAH due to various factors, such as coma, compromised airway protection, risk of aspiration, seizures, elevated ICP, pulmonary dysfunction, and respiratory failure resulting from pre-existing conditions or new complications^37^. The use of PEEP is a crucial component of ventilation strategies in this context. Recent consensus guidelines^38^ suggest that in acute brain injury patients without acute respiratory failure, PEEP levels should be similar to those used in patients without brain injury, and lung protective ventilation strategies can be employed. In patients with both respiratory failure and acute brain injury, lung protective ventilation and higher levels of PEEP may be utilized, as long as clinically significant increases in ICP are not observed. However, there are currently no specific recommendations provided for patients with acute brain injury, respiratory failure and intracranial hypertension^38^.

These consensus guidelines have also recommended targeting a PaO_2_ range of 80–120 mmHg to avoid both hypoxemia and hyperoxia^38^. Positive end-expiratory pressure plays a crucial role in increasing lung functional residual capacity, preventing alveolar de-recruitment, and improving oxygenation^37^. Therefore, increasing PEEP can be a strategy to enhance oxygenation in these patients. Another important consideration in setting PEEP is PbtO_2_, as avoiding cerebral hypoxia, in addition to systemic hypoxemia, is crucial in preventing secondary brain injury in neurocritical care. Additionally, acute lung injury has been associated with brain tissue hypoxia^39^. However, there is limited research on the impact of PEEP on brain oxygenation.

One study involving 20 TBI patients with ARDS demonstrated that increasing PEEP from 5 to 10 to 15 cmH_2_O resulted in significant increases in PbtO_2_ and oxygen saturation, without affecting ICP or CPP in patients without baseline intracranial hypertension^25^. Another study with 10 SAH patients showed that applying PEEP of 20 cmH_2_O resulted in a decrease in PbtO_2_ due to a simultaneous decrease in CPP and cerebral blood flow, which was reversed upon restoration of MAP^27^. Furthermore, a study involving a mixed population of SAH and TBI patients with ARDS demonstrated that recruitment maneuvers and high levels of PEEP significantly improved both systemic and brain tissue oxygenation, leading to reduced oxygen requirements^26^. In our study, increasing PEEP improved PbtO_2_, particularly in patients with low baseline PbtO_2_. Similar responses have been observed with other strategies aimed at enhancing interstitial oxygen availability in acute brain injury patients, such as red blood cell transfusion^40^.

The application of PEEP can have an impact on cardiovascular physiology by increasing intrathoracic pressure; this can potentially decrease venous return, reduce cardiac output, and lead to hypotension, particularly in hypovolemic patients^41^. Hypotension is a significant cause of secondary brain injury, as it can reduce CPP and induce ischemia^42,43^. Previous studies that reported negative effects of PEEP on CPP are typically associated with decreased mean arterial pressure^27,28,44–46^. In our study, both MAP and CPP remained unchanged. It is important to note that increased intrathoracic pressure can lead to decreased cerebral venous drainage, which may elevate ICP^28^. Additionally, higher levels of PEEP and low tidal volumes can result in increased arterial carbon dioxide levels, potentially causing cerebral vasodilation and a subsequent increase in ICP^47^. Consequently, numerous studies have investigated the effects of PEEP on ICP and cerebral hemodynamics.

Wolf et al. demonstrated that employing an open lung strategy with PEEP appears to be safe in patients with acute brain injury and concomitant ARDS, as evidenced by no significant changes in ICP in a small case series (n = 11)^48^. Boone et al. showed minimal effects of PEEP on ICP in patients without severe lung injury, and even in severe lung injury patients, the increase in ICP due to PEEP was considered clinically irrelevant^44^. Other studies conducted in patients with ischemic stroke and TBI have also indicated that the response of ICP to PEEP, although variable, is moderate at best and lacks clinical impact, suggesting that PEEP can be safely utilized in acute brain injury patients when indicated^27,29,45,49–52^. These findings are consistent with our study. However, an older study reported a significant increase in ICP after increments in PEEP among head-injured patients, which had neurological repercussions and necessitated immediate reduction in PEEP^28^.

The impact of PEEP on ICP appears to be influenced by the presence of baseline intracranial hypertension. One study that analyzed changes in ICP after incremental increases in PEEP found that in patients with baseline intracranial hypertension, higher PEEP levels did not affect ICP, while in patients with normal ICP, PEEP levels of 10 and 15 cmH_2_O resulted in increases in ICP without clinical relevance, as CPP remained stable and above 60 mmHg^50^. Other studies have shown that in patients with ICP > 20 mmHg, ICP decreased or remained stable after PEEP increments, while CPP remained unchanged or even increased in some cases^45,52^. In our study, significant increases in ICP due to PEEP increments were observed in 37% (109/295) of episodes, with an ICP exceeding 20 mmHg in 34% (37/109) of episodes. However, baseline ICP was not found to be an independent factor associated with ICP increases following PEEP increments. We were unable to identify any baseline factors that could predict changes in ICP in response to PEEP. Similarly, a recent study also failed to identify baseline factors that could help identify patients at higher risk for ICP increases following PEEP changes^46^.

Another factor that appears to influence ICP responses to variations in PEEP is lung compliance. In patients with low compliance, as is often the case in ARDS patients, higher levels of PEEP had no impact on CPP, ICP or cerebral blood flow^53^. Conversely, in patients with normal compliance, there was a decrease in CPP and cerebral blood flow, but no changes in ICP^45^. A recent study has demonstrated that changes in ICP are inversely correlated with lung recruitability after PEEP application and recruitment maneuvers, suggesting that patients who would benefit from higher PEEP levels will have minimal side effects on intracranial pressure^46^. This can be explained by the fact that the detrimental effects of PEEP are related to alveolar hyperinflation, leading to a significant increase in arterial carbon dioxide and intrathoracic pressure. Conversely, when PEEP leads to alveolar recruitment, improved lung gas distribution, and optimization of ventilation/perfusion matching, ICP remains unchanged^46^. In our study, PaCO_2_ remained stable and within the normal range, which may explain why overall ICP remained unchanged.

Our study has several limitations that should be acknowledged. Firstly, we did not perform a formal sample size calculation, which may have limited the power of our study and prevented us from drawing definitive conclusions. Additionally, this was a single-center study, and therefore, the generalizability of our findings to other centers may be limited. Due to the limited number of events, we were unable to conduct further subgroup analyses, such as comparing patients with baseline tissue hypoxia, baseline intracranial hypertension or differentiating those with ARDS from those without ARDS, which could provide valuable insights into specific patient populations. Another limitation is that we did not specifically assess the correlation between increases in PbtO_2_ after PEEP increments and functional outcomes. Moreover, the retrospective design of our study prevents us from ruling out the potential influence of other concurrent interventions, sedation, or vasopressor therapy, which may have contributed to the observed changes in PbtO_2_ following PEEP increments. Additionally, we lack information on the specific reasons for PEEP changes, as these were determined by the clinical care team. Importantly, we were unable to assess change in respiratory mechanics (such as lung compliance) and correlate them to PbtO_2_ and ICP due to the retrospective nature of this study. Furthermore, the magnitude of PEEP changes in our study was generally small, which could have impacted our results. Moreover, due to how the data was collected (1 h mean) we were unable to perform a time series analysis of the impact of PEEP incrementation on physiological variables. It is also important to note that while PbtO_2_ may assess interstitial oxygen availability, its influence on cellular oxygenation in the brain remains uncertain. Lastly, our findings are not conclusive, as the heterogeneity of our study cohort, which included both traumatic and non-traumatic brain injury patients, and the placement of the probe in hypo perfused cerebral areas (which are more sensitive to interventions aiming to improve oxygen delivery), as well as the use of regional rather than global oxygen monitoring, raise significant issues that should be addressed in future studies on this topic.

## Conclusions

In this study, implementing PEEP to enhance PbtO_2_ could be a viable approach in patients with acute brain injury, particularly those presenting with baseline tissue hypoxia. However, it is crucial to closely monitor ICP and systemic hemodynamics to ensure the safe and appropriate administration of PEEP.

### Supplementary Information

Supplementary Information.

## Data Availability

Due to ethical restrictions, the datasets used and/or analyzed during the current study are available from the corresponding author on reasonable request.

## References

[1] LeRoux P (2014). Consensus summary statement of the international multidisciplinary consensus conference on multimodality monitoring in neurocritical care: A statement for healthcare professionals from the neurocritical care society and the European society of intensive care medicine. Intensive Care Med..

[2] Lazaridis C, Rusin CG, Robertson CS (2019). Secondary brain injury: Predicting and preventing insults. Neuropharmacology.

[3] O'Leary RA, Nichol AD (2018). Pathophysiology of severe traumatic brain injury. J. Neurosurg. Sci..

[4] Taufique Z (2016). Predictors of poor quality of life 1 year after subarachnoid hemorrhage. Neurosurgery.

[5] Lazaridis C, Robertson CS (2016). The role of multimodal invasive monitoring in acute traumatic brain injury. Neurosurg. Clin. N. Am..

[6] Rosengart AJ, Schultheiss KE, Tolentino J, Macdonald RL (2007). Prognostic factors for outcome in patients with aneurysmal subarachnoid hemorrhage. Stroke.

[7] Oddo M (2011). Brain hypoxia is associated with short-term outcome after severe traumatic brain injury independently of intracranial hypertension and low cerebral perfusion pressure. Neurosurgery.

[8] Bardt TF (1998). Monitoring of brain tissue PO_2_ in traumatic brain injury: Effect of cerebral hypoxia on outcome. Acta Neurochir. Suppl..

[9] van den Brink WA (2000). Brain oxygen tension in severe head injury. Neurosurgery.

[10] Maloney-Wilensky E (2009). Brain tissue oxygen and outcome after severe traumatic brain injury: A systematic review. Crit. Care Med..

[11] Kett-White R (2002). Adverse cerebral events detected after subarachnoid hemorrhage using brain oxygen and microdialysis probes. Neurosurgery.

[12] Vath A, Kunze E, Roosen K, Meixensberger J (2002). Therapeutic aspects of brain tissue PO_2_ monitoring after subarachnoid hemorrhage. Acta Neurochir. Suppl..

[13] Chen HI (2011). Detection of cerebral compromise with multimodality monitoring in patients with subarachnoid hemorrhage. Neurosurgery.

[14] Rose JC, Neill TA, Hemphill JC (2006). Continuous monitoring of the microcirculation in neurocritical care: An update on brain tissue oxygenation. Curr. Opin. Crit. Care.

[15] Soehle M, Jaeger M, Meixensberger J (2003). Online assessment of brain tissue oxygen autoregulation in traumatic brain injury and subarachnoid hemorrhage. Neurol. Res..

[16] Haitsma IK, Maas AI (2002). Advanced monitoring in the intensive care unit: brain tissue oxygen tension. Curr. Opin. Crit. Care.

[17] Chesnut RM (1993). The role of secondary brain injury in determining outcome from severe head injury. J. Trauma.

[18] Manley G (2001). Hypotension, hypoxia, and head injury: Frequency, duration, and consequences. Arch. Surg..

[19] Manzano F (2008). Positive-end expiratory pressure reduces incidence of ventilator-associated pneumonia in nonhypoxemic patients. Crit. Care Med..

[20] Neto AS (2016). Epidemiological characteristics, practice of ventilation, and clinical outcome in patients at risk of acute respiratory distress syndrome in intensive care units from 16 countries (PRoVENT): An international, multicentre, prospective study. Lancet Respir. Med..

[21] Yi H (2022). Higher PEEP versus lower PEEP strategies for patients in ICU without acute respiratory distress syndrome: A systematic review and meta-analysis. J. Crit. Care.

[22] Acute Respiratory Distress Syndrome (2000). Ventilation with lower tidal volumes as compared with traditional tidal volumes for acute lung injury and the acute respiratory distress syndrome. N. Engl. J. Med..

[23] Mercat A (2008). Positive end-expiratory pressure setting in adults with acute lung injury and acute respiratory distress syndrome: A randomized controlled trial. JAMA.

[24] Meade MO (2008). Ventilation strategy using low tidal volumes, recruitment maneuvers, and high positive end-expiratory pressure for acute lung injury and acute respiratory distress syndrome: A randomized controlled trial. JAMA.

[25] Nemer SN (2015). Effects of positive end-expiratory pressure on brain tissue oxygen pressure of severe traumatic brain injury patients with acute respiratory distress syndrome: A pilot study. J. Crit. Care.

[26] Wolf S, Plev DV, Trost HA, Lumenta CB (2005). Open lung ventilation in neurosurgery: An update on brain tissue oxygenation. Acta Neurochir. Suppl..

[27] Muench E (2005). Effects of positive end-expiratory pressure on regional cerebral blood flow, intracranial pressure, and brain tissue oxygenation. Crit. Care Med..

[28] Shapiro HM, Marshall LF (1978). Intracranial pressure responses to PEEP in head-injured patients. J. Trauma.

[29] Georgiadis D, Schwarz S, Baumgartner RW, Veltkamp R, Schwab S (2001). Influence of positive end-expiratory pressure on intracranial pressure and cerebral perfusion pressure in patients with acute stroke. Stroke.

[30] Vandenbroucke JP (2007). Strengthening the Reporting of Observational Studies in Epidemiology (STROBE): Explanation and elaboration. PLoS Med..

[31] Carney N (2017). Guidelines for the management of severe traumatic brain injury. Neurosurgery.

[32] Connolly ES (2012). Guidelines for the management of aneurysmal subarachnoid hemorrhage: A guideline for healthcare professionals from the American Heart Association/american Stroke Association. Stroke.

[33] Vincent JL (1996). The SOFA (sepsis-related organ failure assessment) score to describe organ dysfunction/failure on behalf of the working group on sepsis-related problems of the European society of intensive care medicine. Intensive Care Med..

[34] Teasdale G, Jennett B (1974). Assessment of coma and impaired consciousness. A practical scale. Lancet.

[35] Jennett B, Bond M (1975). Assessment of outcome after severe brain damage. Lancet.

[36] Gouvea Bogossian E (2021). Time course of outcome in poor grade subarachnoid hemorrhage patients: A longitudinal retrospective study. BMC Neurol..

[37] Stevens RD, Lazaridis C, Chalela JA (2008). The role of mechanical ventilation in acute brain injury. Neurol. Clin..

[38] Robba C (2020). Mechanical ventilation in patients with acute brain injury: Recommendations of the European Society of Intensive Care Medicine consensus. Intensive Care Med..

[39] Oddo M (2010). Acute lung injury is an independent risk factor for brain hypoxia after severe traumatic brain injury. Neurosurgery.

[40] Gouvea Bogossian E (2022). Factors associated with brain tissue oxygenation changes after RBC transfusion in acute brain injury patients. Crit. Care Med..

[41] Pinsky MR (2005). Cardiovascular issues in respiratory care. Chest.

[42] Chesnut RM (1993). Early and late systemic hypotension as a frequent and fundamental source of cerebral ischemia following severe brain injury in the Traumatic Coma Data Bank. Acta Neurochir. Suppl..

[43] Berthiaume L, Zygun D (2006). Non-neurologic organ dysfunction in acute brain injury. Crit. Care Clin..

[44] Boone MD (2017). The effect of positive end-expiratory pressure on intracranial pressure and cerebral hemodynamics. Neurocrit. Care.

[45] Videtta W (2002). Effects of positive end-expiratory pressure on intracranial pressure and cerebral perfusion pressure. Acta Neurochir. Suppl..

[46] Robba C (2021). Effects of positive end-expiratory pressure on lung recruitment, respiratory mechanics, and intracranial pressure in mechanically ventilated brain-injured patients. Front. Physiol..

[47] Feihl F, Perret C (1994). Permissive hypercapnia: How permissive should we be?. Am. J. Respir. Crit. Care Med..

[48] Wolf S, Schurer L, Trost HA, Lumenta CB (2002). The safety of the open lung approach in neurosurgical patients. Acta Neurochir. Suppl..

[49] Huynh T (2002). Positive end-expiratory pressure alters intracranial and cerebral perfusion pressure in severe traumatic brain injury. J. Trauma.

[50] McGuire G, Crossley D, Richards J, Wong D (1997). Effects of varying levels of positive end-expiratory pressure on intracranial pressure and cerebral perfusion pressure. Crit. Care Med..

[51] Frost EA (1977). Effects of positive end-expiratory pressure on intracranial pressure and compliance in brain-injured patients. J. Neurosurg..

[52] Cooper KR, Boswell PA, Choi SC (1985). Safe use of PEEP in patients with severe head injury. J. Neurosurg..

[53] Burchiel KJ, Steege TD, Wyler AR (1981). Intracranial pressure changes in brain-injured patients requiring positive end-expiratory pressure ventilation. Neurosurgery.

