# Supplementary material for: The effect of increased positive end expiratory pressure on brain tissue oxygenation and intracranial pressure in acute brain injury patients
Source: Sci Rep. 2023 Oct 3;13:16657. doi: 10.1038/s41598-023-43703-9 (PMC10547811; doi:10.1038/s41598-023-43703-9)
Supplement: Supplementary file 1 — Supplementary Information. [file 41598_2023_43703_MOESM1_ESM.docx]

**Supplemental electronic material**

**The effect of increased positive end expiratory pressure on brain tissue oxygenation and intracranial pressure in acute brain injury patients**

*Elisa GOUVEA BOGOSSIAN^1*^MD PhD, Joachim CANTOS^1^ MD,^,^ Anita FARINELLA^1^MD, Leda NOBILE^1^ MD, Hassane NJIMI^1^ Msc PhD, Giacomo COPPALINI^1^MD, Alberto DIOSDADO^1^MD, Michele SALVAGNO^1^MD, Fernando OLIVEIRA GOMES^1^MD, Sophie SCHUIND^2^, MD, Marco ANDERLONI^1^, Chiara ROBBA^3^ MD PhD, Fabio Silvio TACCONE^1^ MD PhD*

*^1^Department of Intensive Care*

*Hôpital Universitaire de Bruxelles (HUB)*

*Université Libre de Bruxelles (ULB)*

*Université Libre de Bruxelles*

*Brussels, Belgium*

*^2^Department of Neurosurgery*

*Hôpital Universitaire de Bruxelles (HUB)*

*Université Libre de Bruxelles (ULB)*

*Université Libre de Bruxelles*

*Brussels, Belgium*

*^3^Dipartimento di Scienze Chirurgiche e Diagnostiche*

*IRCCS Policlinico San Martino*

*Università di Genova*

*Genova, Italy*

**Supplemental table S1:** Univariable generalized mixed model for fixed effects logit link function to assess the impact of baseline variables on the absolute increase in PbtO_2_ after positive end expiratory pressure (PEEP) increments. Data are expressed as odds ratio and 95% confidence intervals. Data from 112 patients with 163 episodes of PEEP incrementation were included in this analysis.

|  | **Univariable analysis**  **OR (95% CI)** | **P value** |
| --- | --- | --- |
| Age, year | 0.99 (0.97-1.02) | 0.59 |
| Male gender | 1.06 (0.52-2.17) | 0.87 |
| Glasgow Coma Scale on admission | 1.0 (0.90-1.11) | 0.98 |
| SOFA score at ICU admission | 0.98 (0.87-1.1) | 0.09 |
| TBI compared to SAH | 2.07 (1.01-4.24) | 0.05 |
| Baseline PEEP | 1.08 (0.95-1.22) | 0.20 |
| Baseline ICP | 0.96 (0.92-1.00) | 0.05 |
| Baseline CPP | 1.01 (0.99-1.03) | 0.28 |
| Baseline PbtO_2_ | 0.95 (0.92-0.97) | 0.001 |
| Baseline PaO_2_ | 1.02 (1.01-1.03) | 0.03 |
| Baseline PaCO_2_ | 1.03 (0.99-1.07) | 0.14 |

SOFA: sequential organ failure assessment; ICU: intensive care unit; TBI: traumatic brain injury; SAH: subarachnoid hemorrhage; PEEP: positive end expiratory pressure; ICP: intracranial pressure; CPP: cerebral perfusion pressure; PbtO_2_: brain tissue partial pressure of oxygen; PaO_2_: arterial partial pressure of oxygen; PaCO_2_: arterial partial pressure of carbon dioxide

**Supplemental table S2:**  Multivariable generalized mixed model for fixed effects logit link function to assess the impact of baseline variables on the absolute increase of brain tissue oxygenation (PbtO_2_) after positive end expiratory pressure (PEEP) increments. Data are expressed as odds ratio and 95% confidence intervals. Data from 112 patients with 163 episodes of PEEP incrementation were included in this analysis.

|  | **Multivariable analysis**  **OR (95% CI)** | **p-value** |
| --- | --- | --- |
| Baseline PaO_2_ | 1.02 (1.01-1.03) | 0.001 |
| Baseline ICP | 0.99 (0.94-1.05) | 0.70 |
| Baseline PbtO_2_ | 0.92 (0.91-0.94) | 0.001 |
| SOFA on ICU admission | 0.97 (0.84-1.12) | 0.67 |
| TBI vs. SAH | 1.65 (0.65-4.19) | 0.29 |

SOFA: sequential organ failure assessment; ICU: intensive care unit; TBI: traumatic brain injury; SAH: subarachnoid hemorrhage; PEEP: positive end expiratory pressure; ICP: intracranial pressure; PbtO_2_: brain tissue partial pressure of oxygen; PaO_2_: arterial partial pressure of oxygen.

**Supplemental Table S3**: Univariable generalized mixed model for fixed effects logit link function to assess the impact of baseline variables on the changes of intracranial pressure (ICP) after positive end expiratory pressure (PEEP) increments. Data are expressed as odds ratio and 95% confidence intervals. Data from 112 patients with 292 episodes of PEEP incrementation were included in this analysis.

|  | **Univariable analysis**  **OR (95% CI)** | **p-value** |
| --- | --- | --- |
| Age, years | 1.01 (0.99-1.02) | 0.50 |
| Male gender | 1.20 (0.74-1.93) | 0.46 |
| Glasgow Coma Scale on admission | 0.99 (0.93-1.06) | 0.06 |
| SOFA score at ICU admission | 1.01 (0.94-1.09) | 0.73 |
| TBI vs. SAH | 0.66 (0.41-1.07) | 0.09 |
| PEEP | 1.02 (0.93-1.12) | 0.66 |
| ICP | 0.97 (0.93-1.01) | 0.10 |
| CPP | 1.01 (0.99-1.02) | 0.30 |
| pH | 1.45 (0.05-44) | 0.83 |
| P/F | 1.00 (0.99-1.002) | 0.91 |
| PaCO_2_ | 1.02 (0.99-1.05) | 0.13 |

SOFA: sequential organ failure assessment; ICU: intensive care unit; TBI: traumatic brain injury; SAH: subarachnoid hemorrhage; PEEP: positive end expiratory pressure; ICP: intracranial pressure; CPP: cerebral perfusion pressure; PbtO_2_: brain tissue partial pressure of oxygen; P/F: arterial partial pressure of oxygen/ inspired fraction of oxygen; PaCO_2_: arterial partial pressure of carbon dioxide

**Supplemental Figure S1:** Correlation matrix analyzed by the Spearman method between changes in PEEP, ICP, PbtO2, CPP and PaO2. Panel A) All measures (N=295). Panel B) First measure per patient (N=112)

**A)**

**
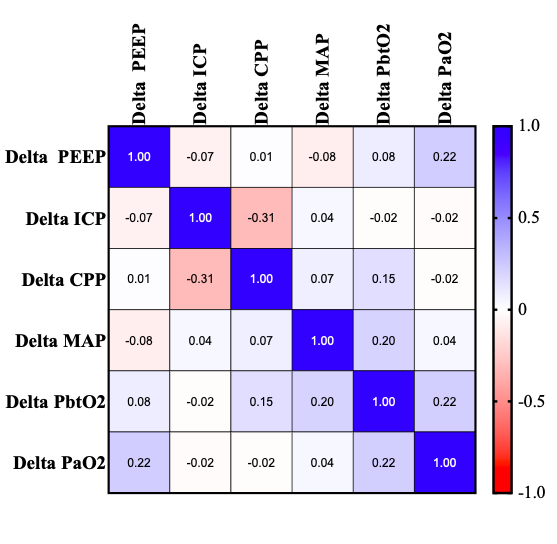
**

**B)**

**
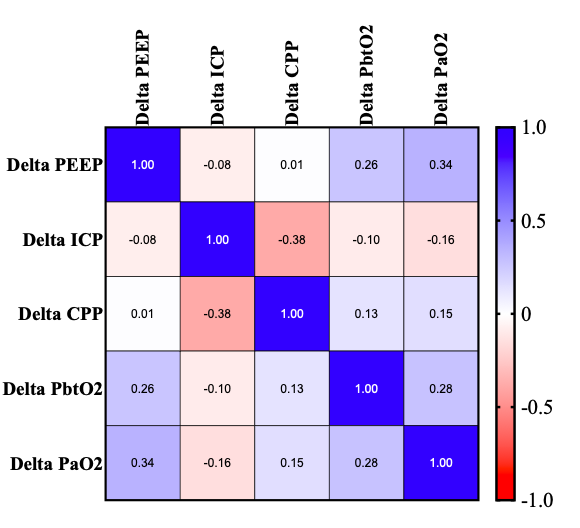
**

**Supplemental Figure S2:** Correlation matrix analyzed by the Spearman method between changes in PEEP, ICP, PbtO2, CPP and PaO2. Panel A) In patients with a significant ICP increase (ICP increase > 20% of baseline or resulting in ICP>20 mmHg) Panel B) In other patients.

**
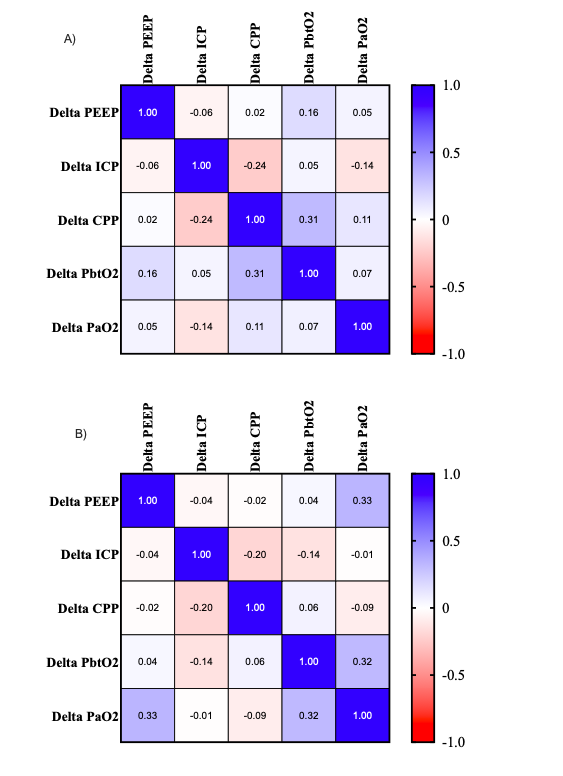
**

**Supplemental Figure S3:** Brain tissue oxygenation - PbtO_2_ (Panel A)and Intracranial pressure - ICP (panel B) changes during PEEP incrementation challenge according to disease etiology (Subarachnoid hemorrhage and traumatic brain.

A)

B)
